# A systematic review and meta-analysis of the efficacy and safety of traditional Chinese medicine in the treatment of rhinosinusitis

**DOI:** 10.1097/MD.0000000000040192

**Published:** 2024-11-29

**Authors:** Zhihao Huang, Xin Xuan, Shan Liu, Jinglan Lin, Zhijun Qian, Lifen Chen, Ruoqing Qiu, Yanwen Cai

**Affiliations:** a Dongguan Hospital of Guangzhou University of Chinese Medicine, Dongguan, Guangdong, China.

**Keywords:** meta-analysis, randomized controlled trial, rhinosinusitis, systematic review, traditional Chinese medicine

## Abstract

**Background:**

Rhinosinusitis (RS), a common inflammatory disorder, adversely affects quality of life and can progress to serious complications without intervention. Conventional therapies, including antibiotics and corticosteroids, exhibit inherent limitations and risks. Traditional Chinese medicine (TCM), with its extensive historical use in RS management, remains understudied in contemporary evaluations. This systematic review evaluates the efficacy and safety of TCM in RS treatment, aiming to guide clinical decision-making.

**Methods:**

A systematic search was undertaken in Chinese and English databases, such as CNKI, Wanfang, VIP, SinoMed, PubMed, Cochrane Library, Web of Science, and other relevant databases, to gather randomized controlled trials of TCM for patients with RS from January 2014 to February 2024. Two researchers independently curated and synthesized data from eligible literature, conducted meta-analyses with ReviewManager 5.4.1, and assessed evidence quality via GRADEPro GDT.

**Results:**

A total of 48 articles included 4490 patients, of which 2249 were control group and 2241 were in the experimental group. Meta-analytic outcomes revealed that the integration of TCM with western medicine conventional treatment significantly enhanced the total effective rate [risk ratio=1.20, 95% confidence interval (CI) (1.17, 1.23), *P* < 0.05] and yielded a notable reduction in Lund–Kennedy score [mean difference (MD)=−1.32, 95% CI (−1.72, −0.93), *P* < 0.05], Lund–Mackay score [MD=−1.13, 95% CI (−1.27, −1.00), *P* < 0.05], SNOT-20 score [MD=−3.02, 95% CI (−4.34, −1.69), *P* < 0.05], nasal congestion Visual Analogue Scale (VAS) score [MD=−1.05, 95% CI (−1.65, −0.45), *P* < 0.05], runny nose VAS score [MD=−0.84, 95% CI (−1.13, −0.54), *P* < 0.05], headache VAS score [MD=−0.90, 95% CI (−1.45, −0.35), *P* < 0.05], olfactory impairment VAS score [MD=−1.43, 95% CI (−1.75, −1.11), *P* < 0.05], and total TCM syndrome score [standardized mean difference (SMD)=−1.78, 95% CI (−2.58, −0.97), *P* < 0.05]. Additionally, significant decreases were observed in levels of tumor necrosis factor-α [SMD=−2.14, 95% CI (−3.42, −0.87), *P* < 0.05] and interleukin-6 [SMD=−1.64, 95% CI (−2.08, −1.21), *P* < 0.05], with statistical significance achieved for all measured outcomes. Regarding safety considerations, an insignificant variance was observed between the 2 therapeutic approaches, with no statistically discernible difference (*P* > 0.05).

**Conclusion:**

Combining TCM with western medicine in RS treatment yields superior outcomes over western medicine alone, with enhanced efficacy, reduced nasal symptoms, and lower inflammation. Rigorous multicenter RCTs are warranted to affirm these advantages and bolster the evidence for TCM in RS management.

## 1. Introduction

Rhinosinusitis (RS) represents an inflammatory disorder involving the nasal cavity and paranasal sinus mucosa, characterized by symptoms such as nasal obstruction, rhinorrhea, headache, and olfactory disturbances. As per the 2020 European Guidelines for the Clinical Care of Rhinosinusitis (EPOS-2020), acute rhinosinusitis (ARS) is categorized as having symptoms that continue for <12 weeks, whereas chronic rhinosinusitis (CRS) is categorized as having symptoms that persist for more than 12 weeks.^[1]^ Global research indicates that the occurrence of RS in adults is expected to range from 5% to 15%, with slightly lower rates in children, ranging from 2% to 12%. In China, the estimated prevalence is around 10%.^[1,2]^ RS exerts a profound influence on the quality of life of affected individuals, potentially culminating in diminished productivity and academic performance. If left untreated or prolonged, it may lead to complications such as asthma and COPD, which can increase the complexity and difficulty of treatment.^[3]^ The causes of RS are varied, including viral infections, bacterial infections, and environmental and allergic factors.^[4]^ Currently, conventional treatment options for RS include antibiotic therapy, topical or systemic corticosteroids, and surgery.^[1]^ However, studies have shown that short-term infections of RS are often caused by viruses, the effectiveness of antibiotic therapy is limited, and the risk of adverse effects must be considered,^[5]^ and the possible side effects of hormone therapy and the risk of recurrence after surgical treatment pose challenges for the treatment of RS. Therefore, the medical community is still exploring and updating the treatment options for patients with RS to seek more effective treatments.

RS is categorized under the “nasal abyss” in the traditional Chinese medical framework, and the treatment of RS by traditional Chinese medicine (TCM) boasts an extensive historical lineage. Classic TCM prescriptions, including Shenling Baizhu San, Gentian Xinggan Soup, and Qianjin Weijing Soup, among others, have demonstrated notable therapeutic efficacy in clinical practice. With the development of modern industry, proprietary Chinese medicines have the advantages of definite efficacy, convenience of taking, and few adverse reactions, and are also widely used in the clinical practice of RS.^[6]^ However, despite the increasing use of TCM in the treatment of RS, there is currently only one systematic review of TCM in the treatment of ARS patients,^[7]^ which does not involve patients with CRS, and the number of included studies is limited, and the evaluation is not comprehensive. The study aims to systematically review and meta-analyze the burgeoning clinical investigations of TCM for RS treatment, providing an exhaustive and unbiased assessment of TCM’s efficacy and safety, thereby establishing an evidence-based foundation to guide clinical practice.

## 2. Information and methods

### 2.1. Inclusion criteria

#### 2.1.1. Study types

Randomized controlled trials (RCTs), unblinded, in Chinese and English.

#### 2.1.2. Research object

Consistent with the clinical diagnosis of RS, age, gender, disease progression, and seriousness of the illness are not limited.

#### 2.1.3. Interventions

The control group received western medicine conventional treatment (WMCT), which encompassed a range of therapeutic approaches. This included the use of western medicine treatments such as nasal corticosteroid sprays, highlighted in the 2020 European Guidelines for the Clinical Treatment of Rhinosinusitis^[1]^ and the 2018 Chinese Guidelines for the Diagnosis and Treatment of Chronic Sinusitis.^[8]^ The specific sprays recommended were mometasone furoate nasal spray, fluticasone propionate nasal spray, and budesonide nasal spray, among others. In addition to these, the WMCT also involved the administration of systemic corticosteroids, including dexamethasone and prednisone, antibiotics like amoxicillin, clarithromycin, and azithromycin, mucus-stimulating agents such as ambroxol hydrochloride and eucalyptus lime enteric-coated capsules, antihistamines (loratadine, azelastine, etc), antileukotriene drugs (montelukast sodium), and nasal decongestants (oxymetazoline hydrochloride, ephedrine nasal drops, etc). Furthermore, the WMCT protocol also included nasal cleansing procedures, utilizing saline nasal spray and nasal irrigation techniques. The experimental group received a combination of TCM treatment and WMCT, where the TCM treatment included oral Chinese patent medicine, classical prescription addition and subtraction, self-simulated formula, etc, not limited to oral dosage form and dose.

#### 2.1.4. Outcome measures

The main end variables included the overall clinical response rate, which encompassed cure, apparent response, and efficacy, while omitting ineffectiveness. Additional outcomes examined were: Lund–Kennedy score, Lund–Mackay score, SNOT-20 score, nasal congestion, runny nose, headache, olfactory impairment, and other visual analogue scale scores (VAS score), total TCM syndrome score, tumor necrosis factor-α (TNF-α), interleukin-6 (IL-6), and incidence of adverse reactions.

### 2.2. Exclusion standards

The exclusion criteria included literature that did not meet the research criteria, such as meta-analyses, reviews, systematic reviews, and animal experiments. Within the interventions, WMCT excluded studies involving surgical procedures, and TCM treatment excluded methods of external Chinese medicine (such as nasal irrigation, nebulization, acupuncture, and massage). For repeated publications, only one article with complete data was included. Additionally, studies were excluded if the full text could not be obtained.

### 2.3. Search strategy

A systematic search was conducted across an array of scholarly databases, including PubMed, Cochrane Library, Web of Science English database, CNKI, Wanfang, VIP, and SinoMed Chinese databases, to identify RCTs involving patients with RS. The search covered the period from January 2014 to February 2024. Chinese search terms: traditional Chinese medicine, granules, powder, soup, rhinosinusitis, acute rhinosinusitis, chronic rhinosinusitis, etc. English search terms: traditional Chinese medicine, Chinese herbal, sinusitis, acute rhinosinusitis, chronic rhinosinusitis, and so on.

### 2.4. Literature screening and data extraction

Two researchers performed independent screening and data extraction in accordance with predefined eligibility criteria and search strategy. In instances of ambiguity or discordance regarding literature selection and data retrieval, resolution was reached through collaborative deliberation or consultation with an additional expert. Using NoteExpress software, we first screened and eliminated duplicate literature, then read remaining literature titles and abstracts, excluded those that didn’t meet the research criteria, read the full texts again, and finally determined the eligibility of the literature and formulated an extraction table. The extracted contents included: first author, publication year, disease type, sample size, sex ratio, age, intervention, comparator, treatment course and outcome measures.

### 2.5. Risk of bias assessment

Two researchers independently assessed the literature quality using the “Risk of Bias” tool from the Cochrane Handbook,^[9]^ and if there were disagreements during the evaluation process, they discussed with a third party to resolve the objections. The review included the following 7 aspects: method of randomization, concealment of randomization scheme, blinding of investigators and participants, blinding of data collectors and analyzers, outcome data completeness, selective reporting of study results, and other sources of bias. The included literature underwent a methodological quality evaluation based on a three-tiered risk of bias classification: low, high, and uncertain across the designated criteria.

### 2.6. Statistical analysis

The statistical software ReviewManager 5.4.1 was used to analyze the data. For dichotomous data, the risk ratio (RR) with a 95% confidence interval (CI) was used as the pooled effect size measure, and for continuous data, the mean difference (MD) or standardized mean difference (SMD) with a 95% CI was used. The chi-square test (at the α level of 0.1) and *I*² statistic were used to assess heterogeneity between studies; a *P* value ≥ 0.1 and *I*² <50% indicated low heterogeneity, prompting the use of a fixed-effect model for meta-analysis, while a *P* value < 0.1 and *I*² ≥50% indicated high heterogeneity, prompting the use of a random-effects model for meta-analysis, subgroup analysis, or sensitivity analysis. For outcomes with more than 10 studies in meta-analysis, publication bias was assessed using funnel plots.

### 2.7. GRADE quality of evidence

Two researchers independently assessed the evidence quality for the outcomes using GRADEPro GDT. They also consulted with a third party to address any issues in the evaluation procedure. The review covered 5 key areas: risk of bias, inconsistency, indirectness, imprecision, and other considerations. The evidence for each outcome was assessed and classified as very low, low, moderate, or high quality.

## 3. Results

### 3.1. Literature screening

**Figure 1.**
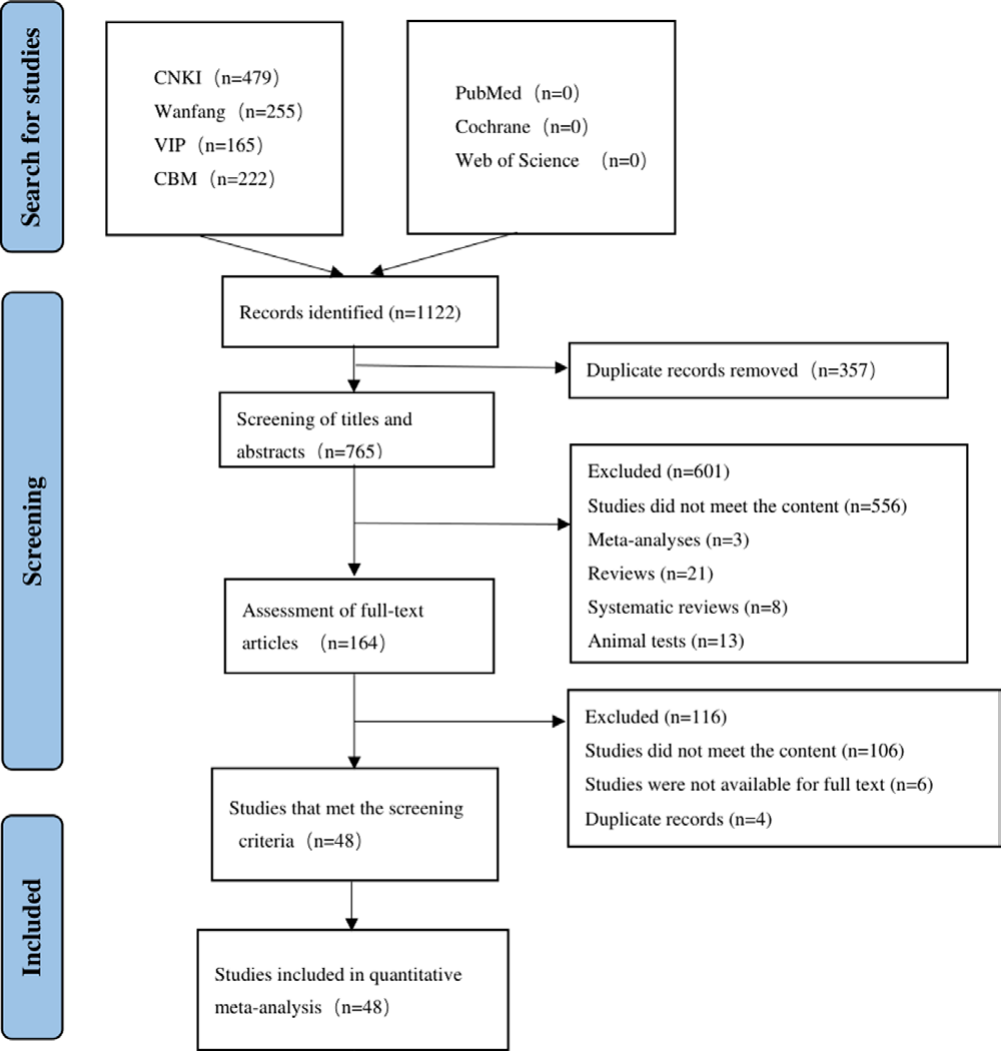
Literature screening process.

The initial screening yielded 1122 documents, from which 357 duplicates were removed using NoteExpress. Subsequent title and abstract review led to the exclusion of 601 articles. Full-text eligibility assessment of the remaining 164 documents resulted in the inclusion of 48 studies that fulfilled the predefined criteria. The screening process is shown in Figure 1.

### 3.2. Basic characteristics of the included literature

The meta-analysis incorporated 48 randomized controlled trials from China, which had comparable baseline characteristics. The total study population consisted of 4425 individuals with RS, with 2216 allocated to the experimental group and 2209 to the control group. Participant numbers ranged from 50 to 236 across the studies. The trials included 15 on ARS^[10–24]^ and 33 on CRS.^[25–57]^ All trials assessed TCM in conjunction with WMCT versus WMCT alone. Key trial features are shown in Table 1, and specific interventions are detailed in Table 2.

**Table 1:** The characteristic of included studies.

Included literature	Type of disease	Sample size/example	Male/female/example		Age/years		Interventions		Course	Outcome measures
			T	C	T	C	T	C		
Chen Juntao (2023) ^[27]^	CRS	40/40	20/20	22/18	11.41 ± 1.65	11.26 ± 1.79	TCM + WMCT	WMCT	28 days	①⑪⑫
Gu Wei (2023)^[28]^	CRS	30/30	16/14	17/13	38.86 ± 5.79	38.15 ± 5.66	TCM + WMCT	WMCT	4 weeks	③④
Xu Shengnan (2023)^[25]^	CRS	60/60	35/25	34/26	43.41 ± 6.28	42.85 ± 6.11	TCM + WMCT	WMCT	12 weeks	①⑦⑧⑪
Li Xiaojuan (2023)^[26]^	CRS	41/41	19/22	21/20	36.51 ± 4.38	35.94 ± 4.58	TCM + WMCT	WMCT	1 month	①⑤⑥⑦⑩⑪
Wang Weiyan (2023)^[10]^	ARS	48/48	25/23	26/22	48.59 ± 5.80	48.63 ± 5.76	TCM + WMCT	WMCT	10 days	①⑨
Liu Ying (2022)^[31]^	CRS	68/68	39/29	35/33	41.4 ± 10.3	39.9 ± 8.8	TCM + WMCT	WMCT	12 weeks	①②③④⑤⑥⑫
Sun Yi (2022)^[29]^	CRS	30/30	19/11	17/13	44.72 ± 4.31	44.81 ± 4.24	TCM + WMCT	WMCT	12 weeks	①⑩⑪⑫
Chen Jing (2022)^[30]^	CRS	50/50	26/24	27/23	31.2 ± 10.5	31.1 ± 10.6	TCM + WMCT	WMCT	2 months	①②
Wu Yumei (2021)^[35]^	CRS	31/31	14/17	15/16	45.4 ± 1.5	45.1 ± 2.6	TCM + WMCT	WMCT	4 weeks	①⑫
Hao Yanan (2022)^[14]^	ARS	60/60	26/34	29/31	35.22 ± 6.71	35.34 ± 6.58	TCM + WMCT	WMCT	15 days	①②⑨⑩⑪⑫
Zheng Yongfeng (2022)^[15]^	ARS	37/36	24/13	21/15	5.89 ± 2.42	6.69 ± 2.31	TCM + WMCT	WMCT	14 days	①⑫
Wang Li (2022)^[12]^	ARS	45/45	26/19	22/23	9.15 ± 1.23	8.97 ± 1.42	TCM + WMCT	WMCT	14 days	①②
Chen Xiaoxia (2022)^[13]^	ARS	118/118	62/56	65/53	7.08 ± 1.55	7.05 ± 1.43	TCM + WMCT	WMCT	4 days	①⑫
Zhang Jingyi (2022)^[11]^	ARS	50/50	16/34	17/33	43.25 ± 7.65	44.16 ± 7.56	TCM + WMCT	WMCT	7 days	①③⑫
Tong Zhao Quan (2021)^[32]^	CRS	42/42	26/16	24/18	28.44 ± 10.37	28.54 ± 10.24	TCM + WMCT	WMCT	2 weeks	①
Qi Tongfei (2021)^[33]^	CRS	37/36	20/17	21/15	46.14 ± 10.05	44.75 ± 9.37	TCM + WMCT	WMCT	2 months	①②③⑤⑥⑧⑨
Pang Shuai (2021)^[34]^	CRS	55/55	29/26	30/25	7.74 ± 2.69	8.36 ± 2.67	TCM + WMCT	WMCT	1 month	①
He Zaodi (2021)^[16]^	ARS	40/40	23/17	21/19	34.93 ± 11.38	35.30 ± 11.46	TCM + WMCT	WMCT	1 week	①⑨⑩
Ahmat Mukhtar (2020)^[39]^	CRS	30/30	8/20	14/13	37.64 ± 12.93	38.89 ± 13.65	TCM + WMCT	WMCT	4 weeks	①
Tang Nan (2020)^[37]^	CRS	40/40	23/17	22/18	39.5 ± 17.5	40.5 ± 17.5	TCM + WMCT	WMCT	8 weeks	①②④
Meng Yajun (2020)^[38]^	CRS	36/36	25/11	24/12	－	－	TCM + WMCT	WMCT	3 weeks	①⑨
Yu Fengying 2020^[18]^	ARS	51/55	34/17	31/24	8.6 ± 3.2	6.8 ± 2.5	TCM + WMCT	WMCT	2 weeks	①⑩⑫
Zhong Minru (2020)^[17]^	ARS	56/56	29/27	31/25	36.19 ± 6.45	35.24 ± 6.73	TCM + WMCT	WMCT	7 days	①②⑨⑩⑪
Cai Chujun (2019)^[42]^	CRS	28/27	8/20	14/13	44.14 ± 10.18	44.74 ± 12.26	TCM + WMCT	WMCT	28 days	①②④⑫
Li Weili (2019)^[43]^	CRS	60/60	38/22	36/24	38.67 ± 3.35	39.25 ± 3.43	TCM + WMCT	WMCT	90 days	①②③
Pan Chengjun (2021)^[36]^	CRS	38/38	20/18	22/16	50.2 ± 4.9	49.3 ± 4.7	TCM + WMCT	WMCT	8 weeks	①②⑤⑥
Xu Wei (2019)^[40]^	CRS	43/43	24/19	26/17	38.8 ± 9.4	38.2 ± 9.1	TCM + WMCT	WMCT	8 weeks	①⑤⑥⑧
Chen Bo (2019)^[41]^	CRS	38/38	27/11	25/13	7.33 ± 3.06	7.24 ± 3.15	TCM + WMCT	WMCT	2 weeks	①②
Han Ruihua (2018)^[44]^	CRS	50/50	23/27	27/23	55.5 ± 5.6	55.1 ± 5.3	TCM + WMCT	WMCT	12 weeks	①②③⑫
Zheng Haiming (2018)^[45]^	CRS	38/37	17/21	17/20	26.8 ± 1.7	27.3 ± 2.6	TCM+ WMCT	WMCT	45 days	①
Chen Cuicui (2018)^[46]^	CRS	58/58	35/23	33/25	47.5 ± 2.8	47.3 ± 2.6	TCM+ WMCT	WMCT	2 weeks	①⑩
Hou Linlin (2018)^[47]^	CRS	36/36	25/11	26/10	38.12 ± 4.14	38.17 ± 4.11	TCM + WMCT	WMCT	30 days	①⑫
Zhang Yang (2018)^[48]^	CRS	50/50	28/22	26/24	48.23 ± 8.71	49.56 ± 9.13	TCM + WMCT	WMCT	4 weeks	①
Yue Yanqin (2018)^[21]^	ARS	32/32	21/11	22/10	7 ± 2.52	9 ± 3.31	TCM + WMCT	WMCT	15 days	①
Yang Chunling (2018)^[20]^	ARS	41/41	25/16	23/18	5.84 ± 2.36	5.72 ± 2.29	TCM + WMCT	WMCT	7–14 days	①
Yang Juan (2018)^[19]^	ARS	25/25	13/12	15/10	41.05 ± 2.16	41.68 ± 2.95	TCM + WMCT	WMCT	1 week	①
Zhu Xiaopu (2017)^[50]^	CRS	48/48	27/21	25/23	35.32 ± 5.42	35.20 ± 5.80	TCM + WMCT	WMCT	4 weeks	①⑫
Du Jingwei (2016)^[51]^	CRS	44/40	25/19	23/17	32.25 ± 9.48	33.02 ± 9.35	TCM + WMCT	WMCT	20 days	②⑩⑪⑫
Huang Yanchang (2016)^[54]^	CRS	39/39	20/19	19/20	29 ± 5.83	29 ± 6.77	TCM + WMCT	WMCT	4 weeks	①
Yang Guizhen (2016)^[52]^	CRS	48/48	－	－	－	－	TCM + WMCT	WMCT	2 weeks	①
Cao Gang (2016)^[53]^	CRS	30/30	－	－	－	－	TCM + WMCT	WMCT	1 month	①⑫
Su Jinhui (2016)^[22]^	ARS	50/50	30/20	29/21	43.71 ± 12.41	43.67 ± 12.37	TCM + WMCT	WMCT	7 days	①
Shi Fenglei (2016)^[23]^	ARS	33/32	－	－	－	－	TCM + WMCT	WMCT	7 days	①
Quan Meiyu (2017)^[49]^	CRS	70/70	40/30	36/34	38.7 ± 2.5	39.1 ± 2.1	TCM + WMCT	WMCT	7–14 days	①
Chen Xiangjun (2015)^[55]^	CRS	99/99	－	－	－	－	TCM + WMCT	WMCT	－	①
Jiang Liyuan (2015)^[56]^	CRS	36/36	16/20	24/12	42.6 ± 3.5	40.2 ± 4.5	TCM + WMCT	WMCT	9 weeks	①⑫
Yu Fengci (2014)^[57]^	CRS	45/43	25/20	24/19	7.68 ± 2.60	8.17 ± 3.05	TCM + WMCT	WMCT	1–4 weeks	①
Chen Guoxiang (2014)^[24]^	ARS	42/42	27/15	20/22	－	－	TCM + WMCT	WMCT	7–15 days	①

*Notes*: T: experimental group, C: control group, –: not reported. ① Effective rate, ② Lund–Kennedy score, ③ Lund–Mackay score, ④ SNOT-20 score, ⑤ nasal congestion VAS score, ⑥ runny nose VAS score, ⑦ headache VAS score, ⑧ olfactory impairment VAS score, ⑨ TCM syndrome total score, ⑩ TNF-α, ⑪ IL-6, ⑫ adverse reactions and safety evaluation.

ARS = acute rhinosinusitis, CRS = chronic rhinosinusitis, TCM = traditional Chinese medicine, WMCT = western medicine conventional treatment.

**Table 2:** Specific interventions.

Included literature	TCM formula	TCM dosage (amount, frequency)	TCM route of administration	TCM treatment duration	WMCT
Chen Juntao (2023)^[27]^	Xin Zhi Bi Kang Decoction	100 mL, bid	Oral	28 days	Triamcinolone nasal spray + loratadine syrup
Gu Wei (2023)^[28]^	Bi Yuan Decoction	200 mL, bid	Oral	4 weeks	Clindamycin capsules + furol nasal drops + nasal irrigation
Xu Shengnan (2023)^[25]^	Xiangju Capsules	0.3 g, tid	Oral	12 weeks	Eucalyptus carabinieri enteric soft capsule
Li Xiaojuan (2023)^[26]^	Bi Yuan Prescription	200 mL, bid	Oral	1 month	Clarithromycin dispersible tablets
Liu Ying (2022)^[31]^	Qi-Opening Decoction	200 mL, bid	Oral	12 weeks	Mometasone furoate nasal spray + nasal irrigation
Sun Yi (2022)^[29]^	Bi Yuan Tong Qiao Prescription	200 mL, bid	Oral	12 weeks	Mometasone furoate nasal spray
Chen Jing (2022)^[30]^	Self-prescribed Chinese Herbal Formula	1 dose, bid	Oral	2 months	Azithromycin tablets + mometasone furoate nasal spray
Wu Yumei (2021)^[35]^	Spleen-Strengthening and Dampness-Transforming Qi-Opening Decoction	200 mL, bid	Oral	4 weeks	Clarithromycin
Tong Zhaoquan (2021)^[32]^	Yin Hua Tong Qiao Bi Yan Tang	200 mL, bid	Oral	2 weeks	Penicillin sodium + ephedrine nasal drops
Qi Tongfei (2021)^[33]^	Yang Yin Qing Fei Tang	100 mL, bid	Oral	2 months	Fluticasone propionate nasal spray + eucalyptus carabinieri enteric soft capsule
Pang Shuai (2021)^[34]^	Bi Yuan Tong Qiao Granules	7.5–15 g, tid	Oral	1 month	Amoxicillin capsules + fluticasone propionate nasal spray
Ahmat Mukhtar (2020)^[39]^	Wen Fei Zhi Liu Dan	250 mL, qd	Oral	28 days	Mometasone furoate nasal spray
Tang Nan (2020)^[37]^	Long Dan Xie Gan Tang	200 mL, bid	Oral	12 weeks	Azithromycin capsules + mometasone furoate nasal spray
Meng Yajun (2020)^[38]^	Shen Ling Bai Zhu San He Cang Er Zi San	200 mL, bid	Oral	3 weeks	Cefminox sodium
Cai Chujun (2019)^[42]^	Gan Lu Xiao Du Dan	200 mL, bid	Oral	28 days	Mometasone furoate nasal spray
Li Weili (2019)^[43]^	Long Dan Xie Gan Tang	200 mL, tid	Oral	90 days	Mometasone furoate nasal spray + clarithromycin extended-release tablets
Pan Chengjun (2021)^[36]^	Qing Re Xie Zhuo Tong Qiao Tang	200 mL, bid	Oral	8 weeks	Budesonide nasal spray + clarithromycin tablets + eucalyptus carabinieri enteric soft capsule
Xu Wei (2019)^[40]^	Bi Yuan Tong Qiao Tang	150 mL, bid	Oral	8 weeks	Clarithromycin tablets + fluticasone propionate nasal spray
Chen Bo (2019)^[41]^	Qing Xuan Bi Qiao Yin	1 dose, qd	Oral	2 weeks	Cefdinir capsules
Han Ruihua (2018)^[44]^	Self-prescribed Chinese Herbal Formula	150 mL, bid	Oral	4 weeks	Azithromycin + fluticasone propionate nasal spray + eucalyptus carabinieri enteric soft capsule + montelukast
Zheng Haiming (2018)^[45]^	Self-prescribed Chinese Herbal Formula	1 dose, bid	Oral	45 days	Moxifloxacin + cefdinir
Chen Cuicui (2018)^[46]^	Tong Qiao Bi Yan Prescription	200 mL, bid	Oral	2 weeks	Roxithromycin capsules + oxymetazoline nasal drops
Hou Linlin (2018)^[47]^	Self-prescribed Chinese Herbal Formula	1 dose, bid	Oral	30 days	Amoxicillin + triamcinolone nasal spray
Zhang Yang (2018)^[48]^	Long Dan Xie Gan Tablets	8 pills, bid	Oral	4 weeks	Acetylspiramycin tablets + triamcinolone nasal spray + eucalyptus carabinieri enteric soft capsule
Zhu Xiaopu (2017)^[50]^	Xing Qiao Tang	250 mL, bid	Oral	28 days	Budesonide nasal spray + amoxicillin clavulanate + saline nasal spray
Du Jingwei (2016)^[51]^	Sinusitis Oral Liquid	10 mL,tid	Oral	20 days	Clarithromycin capsules
Huang Yanchang (2016)^[54]^	Bu Fei Yi Pi Tang	150 mL, bid	Oral	4 weeks	Unspecified
Yang Guizhen (2016)^[52]^	Self-prescribed Chinese Herbal Formula	1 dose, bid	Oral	2 weeks	Moxifloxacin + cefdinir
Cao Gang (2016)^[53]^	Self-prescribed Chinese Herbal Formula	1 dose, bid	Oral	1 month	Azithromycin dry suspension + loratadine dispersible tablets
Quan Meiyu (2017)^[49]^	Self-prescribed Chinese Herbal Formula	100 mL, tid	Oral	7–14 days	Roxithromycin capsules + dexamethasone tablets + fluticasone propionate nasal spray
Chen Xiangjun (2015)^[55]^	Yu Ping Feng San	1 bag, tid	Oral	–	Cefoperazone tablets
Jiang Liyuan (2015)^[56]^	Tuo Li Xiao Du San	1 dose, bid	Oral	9 weeks	Clarithromycin tablets
Yu Fengci (2014)^[57]^	Shen Ling Bai Zhu San He Cang Er Zi San	1 dose, qd	Oral	1–4 weeks	Cefprozil granules + furol nasal drops + loratadine tablets
Wang Weiyan (2023)^[10]^	Qian Jin Wei Jing Tang	1 dose, bid	Oral	10 days	Budesonide nasal spray
Hao Yanan (2022)^[14]^	Wind-Dispersing and Qi-Opening Drop Pills	20 pills, tid	Oral	15 days	Cefixime dispersible tablets
Zheng Yongfeng (2022)^[15]^	Xiangju Capsules	1–2 capsules, bid/tid	Oral	14 days	Cefadroxil tablets
Wang Li (2022)^[12]^	Qing Re Xuan Bi Tang	1/3–1 bags, bid	Oral	14 days	Cefpodoxime proxetil for suspension
Chen Xiaoxia (2022)^[13]^	Wind-Dispersing and Qi-Opening Drop Pills	5–10 pills, tid	Oral	4 days	Amoxicillin and clavulanate potassium tablets
Zhang Jingyi (2022)^[11]^	Xin Qian Gan Ju Tang	150 mL, bid	Oral	7 days	Cefuroxime axetil tablets + mometasone furoate nasal spray
He Zaodi (2021)^[16]^	Xin Zhi Tong Qiao Prescription	200 mL, bid	Oral	1 month	Cefprozil dispersible tablets
Yu Fengying (2020)^[18]^	Self-prescribed Chinese Herbal Formula	1 dose, bid	Oral	2 weeks	Amoxicillin clavulanate/clarithromycin
Zhong Minru (2020)^[17]^	Huang Qin Hua Shi Tang	250 mL, bid	Oral	7 days	Cefuroxime axetil tablets
Yue Yanqin (2018)^[21]^	Cang Er Zi San	1 dose, qd	Oral	15 days	Clarithromycin + saline nasal spray/nasal irrigation
Yang Chunling (2018)^[20]^	Qing Re Hua Zhuo Tang	50–100 mL, bid	Oral	7–14 days	Penicillin sodium + cefuroxime sodium + oxymetazoline nasal spray + ambroxol oral solution
Yang Juan (2018)^[19]^	Xuan Fei Tong Qiao Granules	1 dose, bid	Oral	1 week	Amoxicillin
Su Jinhui (2016)^[22]^	Sinusitis Soft Capsules	3–4 capsules, tid	Oral	7 days	Cefuroxime sodium
Shi Fenglei (2016)^[23]^	Xuan Fei Tong Qiao Granules	1 bag, bid	Oral	7 days	Amoxicillin + furol nasal drops
Chen Guoxiang (2014)^[24]^	Shen Ling Bai Zhu San	1 dose, bid	Oral	15 days	Clarithromycin

*Note*: –: not reported.

TCM = traditional Chinese medicine, WMCT = western medicine conventional treatment.

### 3.3. Risk of bias assessment

**Figure 2.**
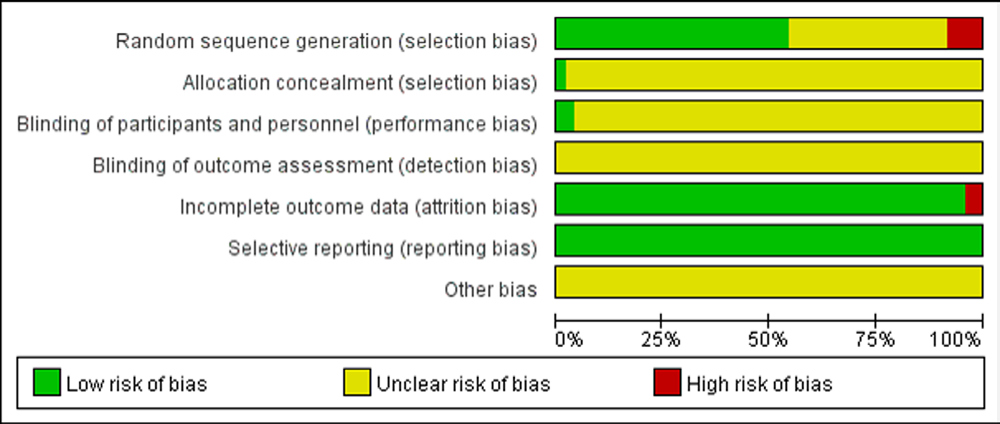
Risk of bias graph.

**Figure 3.**
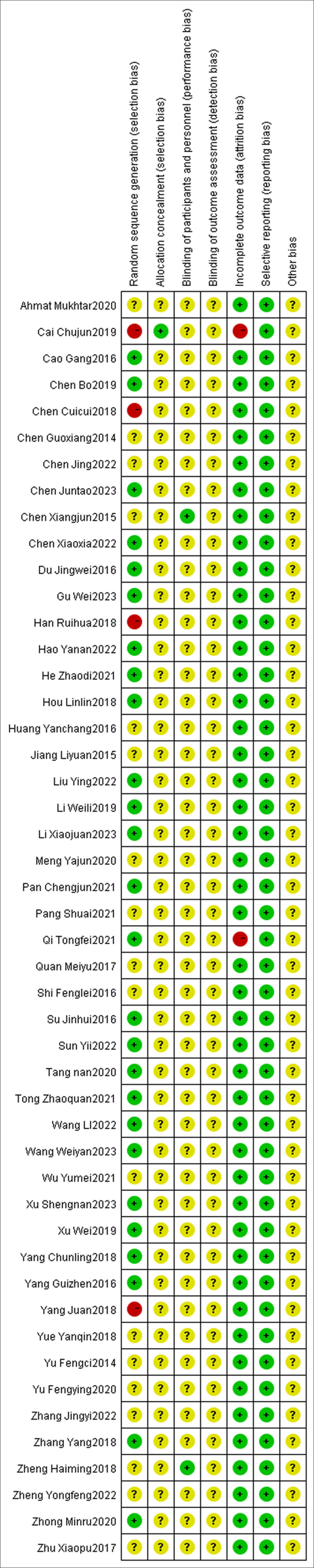
Risk of bias summary.

The risk of bias across the included studies was evaluated using the assessment instrument outlined in the Cochrane Handbook. Of the 48 articles analyzed, 23 utilized random number tables for randomization,^[10,12,14,16,17,20,25–27,29,31–33,36,37,40,41,43,47,48,51–53]^ 1 used computer randomization,^[28]^ 1 used random permutation,^[13]^ 1 used digital lots,^[22]^ and 11 articles mentioned randomization without providing details about the method.^[11,23,24,30,34,35,38,39,49,50,54]^ In 7 instances,^[15,18,21,45,55–57]^ randomization was not mentioned, leaving the risk of bias unknown. Three items^[42,44,46]^ were grouped based on presentation order, one^[19]^ on odd and even numbers, and these were classified as high risk. One item^[42]^ was repeated. Allocation concealment using the random envelope method was evaluated as low risk; none of the other studies described allocation concealment, leaving the risk unknown. Two studies^[45,55]^ implemented double-blinding of implementers and participants, assessed as low risk; the rest did not mention blinding, with the risk remaining unknown. No studies mentioned blinding of outcome assessment, with the risk also unknown. Two studies^[33,42]^ reported dropout rates and their causes, but did not perform intention-to-treat analysis, evaluated as high risk; the rest had complete outcome data, rated as low risk. All studies listed predetermined outcome measures, assessed as low risk. Insufficient data was present in all studies to assess additional biases, with the risk unknown. See Figures 2 and 3 for details.

### 3.4. Meta-analysis

#### 3.4.1. Total effective rate

**Figure 4.**
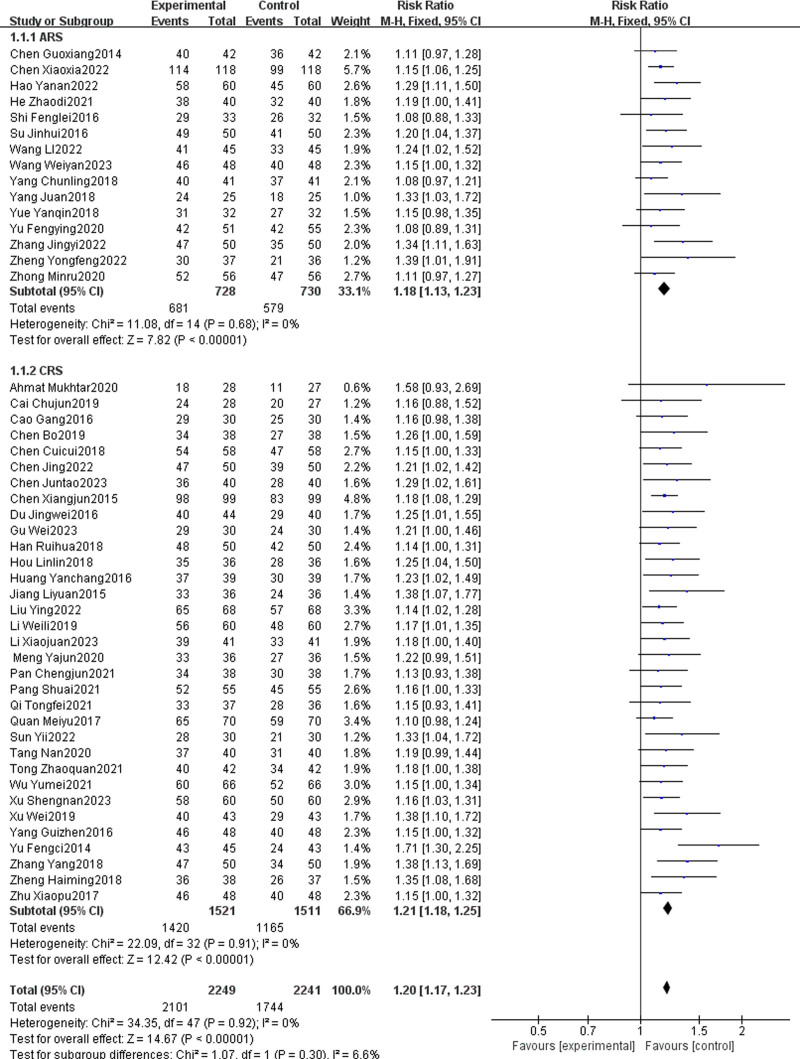
Meta-analysis of total effective rate.

Total effective rate is defined as the proportion of patients who achieved either clinical cure or significant improvement following treatment. Forty-eight articles^[10–57]^ reported the total effective rate of TCM combined with WMCT, involving a total of 4490 patients. Among these, 15 articles focused on ARS, and 33 articles focused on CRS. The heterogeneity assessment (*P* = .92, *I*² = 0%) indicated no significant variability among studies, with the experimental group showing a significantly higher total effective rate compared to the control group [RR = 1.20, 95% CI (1.17, 1.23), *P* < .00001, Fig. 4]. Subgroup analysis by disease type revealed that for ARS, the relative risk was [RR = 1.18, 95% CI (1.13, 1.23), *P* < .00001], and for CRS, the relative risk was [RR = 1.21, 95% CI (1.18, 1.25), *P* < .00001].

#### 3.4.2. Lund–Kennedy score

**Figure 5.**
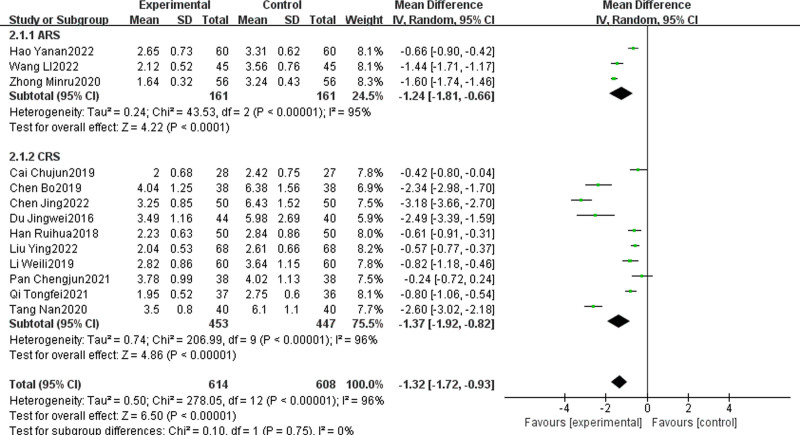
Meta-analysis of Lund–Kennedy scores.

Thirteen articles^[11,12,14,17,30,31,33,36,37,41–44,51]^ examined the Lund–Kennedy score of TCM + WMCT compared to WMCT. The collective study cohort across these articles comprised 1222 participants. Among these, 3 articles focused on ARS, and 10 articles focused on CRS. The findings revealed a statistically significant reduction in Lund–Kennedy scores for the experimental group in comparison to the control group [MD = ‐1.32, 95% CI: ‐1.72 to ‐0.93, *P* < .00001, Fig. 5]. Subgroup analysis outcomes demonstrated that the experimental group achieved a more pronounced reduction in Lund–Kennedy scores compared to the control group for both ARS [MD = ‐1.24, 95% CI ‐1.81 to ‐0.66, *P* < .0001] and CRS [MD = ‐1.37, 95% CI ‐1.92 to ‐0.82, *P* < .00001].

#### 3.4.3. Lund–Mackay score

**Figure 6.**
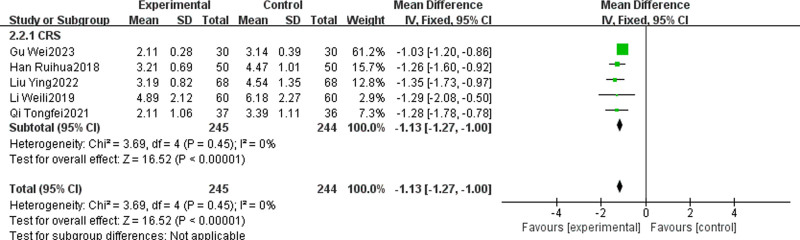
Meta-analysis of Lund–Mackay scores.

Several studies^[28,31,33,43,44]^ have compared the Lund–Mackay score of TCM + WMCT to the score of WMCT alone. The studies had a total of 489 patients. All patients had CRS. The findings indicated a more pronounced reduction in Lund–Mackay scores for the experimental group compared to the control [MD = ‐1.13, 95% CI (‐1.27, ‐1.00), *P* < .00001, Fig. 6].

#### 3.4.4. SNOT-20 score

**Figure 7.**
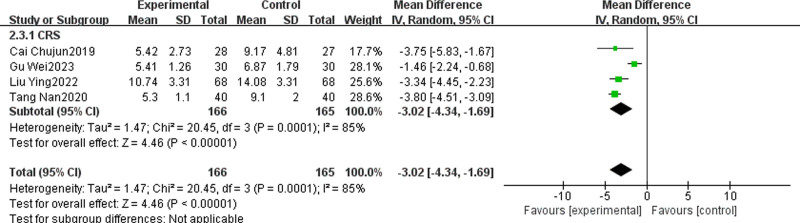
Meta-analysis of SNOT-20 scores.

Four studies^[28,31,37,42]^ reported the SNOT-20 score of TCM + WMCT versus WMCT, involving 331 patients, all of whom were CRS. The data revealed significantly greater reductions in SNOT-20 scores for the experimental group versus the control [MD = ‐3.02, 95% CI (‐4.34, ‐1.69), *P* < .00001, Fig. 7].

#### 3.4.5. Nasal congestion VAS score

**Figure 8.**
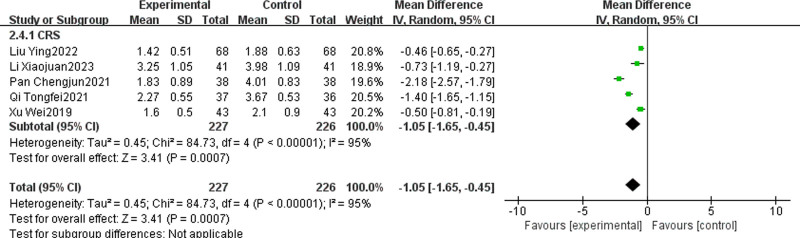
Meta-analysis of nasal congestion VAS score. VAS = visual analogue scale.

Five studies^[26,31,33,36,40]^ reported the VAS score of nasal congestion in TCM + WMCT versus WMCT, involving 453 patients, all of whom were CRS. The results indicated a pronounced reduction in the VAS score for nasal congestion in the experimental group over the control [MD = ‐1.05, 95% CI (‐1.65, ‐0.45), *P* = .0007, Fig. 8].

#### 3.4.6. Runny nose VAS score

**Figure 9.**
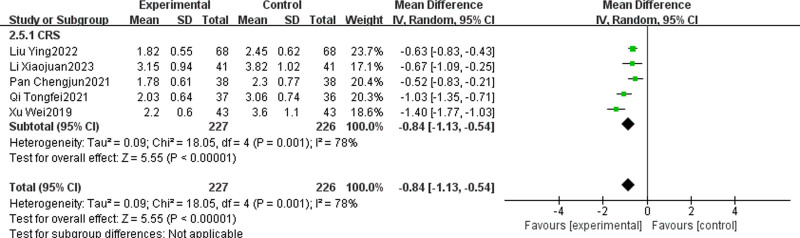
Meta-analysis of runny nose VAS score. VAS = visual analogue scale.

Five articles^[26,31,33,36,40]^ reported the VAS score of TCM + WMCT versus WMCT, involving 453 patients, all of which were CRS. The data revealed that the experimental group achieved a more substantial decrease in nasal congestion scores than the control [MD = ‐0.84, 95% CI (‐1.13, ‐0.54), *P* < .00001, Fig. 9].

#### 3.4.7. Headache VAS score

**Figure 10.**
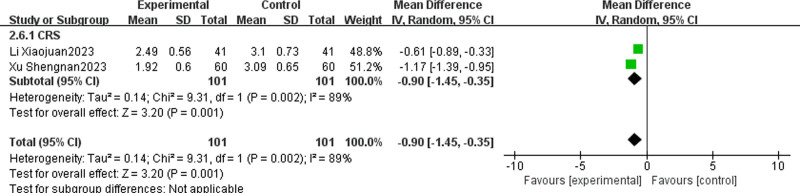
Meta-analysis of headache VAS score. VAS = visual analogue scale.

Two studies^[25,26]^ compared the headache VAS scores of patients receiving TCM + WMCT to those receiving WMCT. The collective participant pool across the studies encompassed 202 individuals, all diagnosed with CRS. The data indicated a marked reduction in headache VAS scores for the experimental group compared to the control [MD = ‐0.90, 95% CI (‐1.45, ‐0.35), *P* = .001, Fig. 10].

#### 3.4.8. Olfactory impairment VAS score

**Figure 11.**
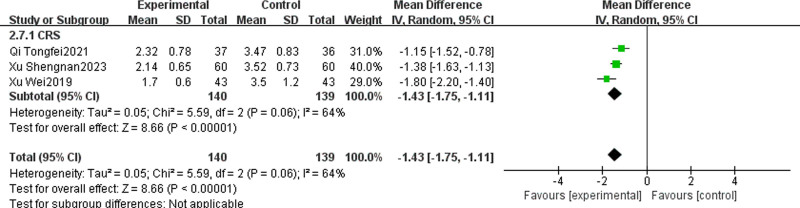
Meta-analysis of VAS score for olfactory impairment. VAS = visual analogue scale.

Three studies^[25,33,40]^ compared the VAS scores for olfactory impairment between the TCM + WMCT group and the WMCT group. The studies involved a total of 279 patients with CRS. The data suggested a significant enhancement in olfactory impairment VAS score reduction for the experimental group over the control [MD = ‐1.43, 95% CI (‐1.75, ‐1.11), *P* < .00001, Fig. 11].

#### 3.4.9. Total TCM syndrome score

**Figure 12.**
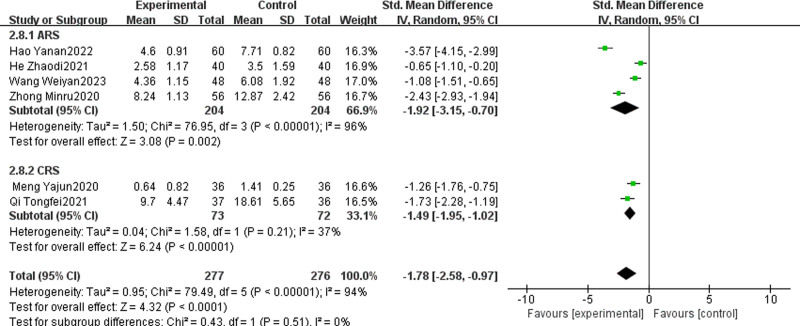
Meta-analysis of TCM syndrome total scores. TCM = traditional Chinese medicine.

Six studies, comprising 4 focused on ARS and 2 on CRS, were identified for examination.^[10,14,16,17,33,38]^ These studies included a total of 553 patients. The combined analysis of these studies showed that the experimental group, which received both TCM and WMCT, demonstrated superior efficacy in reducing the total TCM syndrome score compared to the control group, which received WMCT alone [SMD = ‐1.78, 95% CI (‐2.58, ‐0.97), *P* < .00001, Fig. 12]. Further subgroup analysis highlighted that the experimental group had a more significant reduction in the total TCM syndrome score for both ARS [SMD = ‐1.92, 95% CI (‐3.15, ‐0.70), *P* = .002] and CRS [SMD = ‐1.49, 95% CI (‐1.95, ‐1.02), *P* < .00001].

#### 3.4.10. Tumor necrosis factor-α

**Figure 13.**
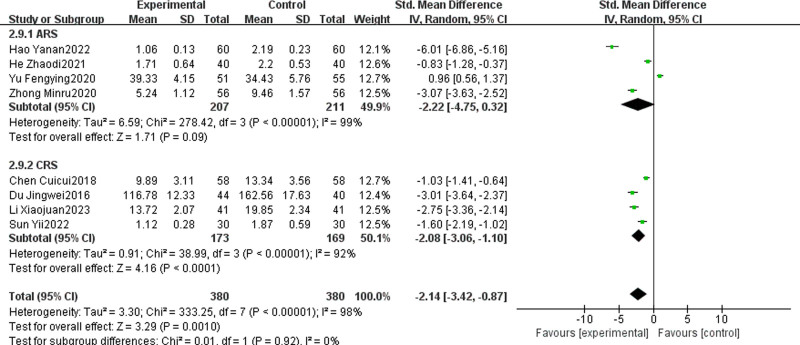
Meta-analysis of TNF-α. TNF-α = tumor necrosis factor-α.

Eight studies, 4 on ARS and 4 on CRS, compared TCM + WMCT to WMCT alone, involving 760 patients.^[14,16–18,26,29,46,51]^ The experimental group showed a significantly greater reduction in TNF-α levels than the control group [SMD = ‐2.14, 95% CI (‐3.42, ‐0.87), *P* = .001, Fig. 13]. For ARS, the experimental group’s reduction in TNF-α was not significantly different from the control group [SMD = ‐2.22, 95% CI (‐4.75, 0.32), *P* = .09]. In contrast, for CRS, the experimental group had a significantly greater reduction in TNF-α levels compared to the control group [SMD = ‐2.08, 95% CI (‐3.06, ‐1.10), *P* < .0001].

#### 3.4.11. Interleukin-6

**Figure 14.**
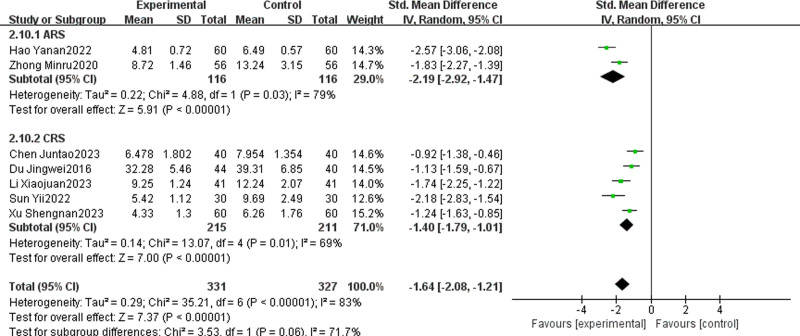
Meta-analysis of IL-6. IL-6 = interleukin-6.

Seven articles, comprising 2 on ARS and 5 on CRS, evaluated the effect of combining TCM with WMCT on IL-6 levels.^[14,17,25–27,29,51]^ These studies included a total of 658 patients. The experimental group, receiving TCM plus WMCT, showed a significant reduction in IL-6 levels compared to the control group, which received only WMCT [SMD = ‐1.64, 95% CI (‐2.08, ‐1.21), *P* < .00001, Fig. 14]. Subgroup analysis revealed that the experimental group was significantly more effective in reducing IL-6 levels in both ARS [SMD = ‐2.19, 95% CI (‐2.92, ‐1.47), *P* < .00001] and CRS [SMD = ‐1.40, 95% CI (‐1.79, ‐1.01), *P* < .00001] when compared to their respective control groups.

#### 3.4.12. Adverse reactions

**Figure 15.**
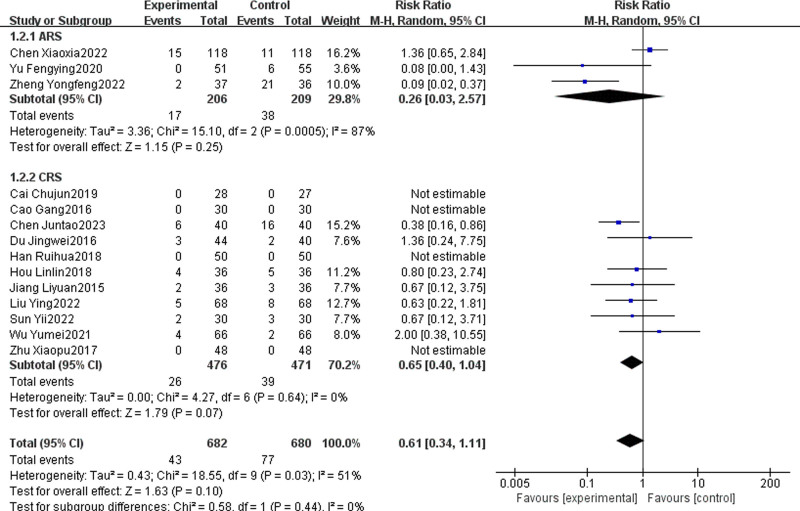
Meta-analysis of the incidence of adverse reactions.

Fourteen articles, with 3 focusing on ARS and 11 on CRS, reported on the adverse reactions associated with the combination of TCM and WMCT versus WMCT alone.^[13,15,18,27,29,31,35,42,44,47,50,51,53,56]^ These studies included a total of 1362 patients. The overall results showed no significant difference in the occurrence of adverse reactions between the experimental group and the control group [RR = 0.61, 95% CI (0.40, 1.04), *P* = .10, Fig. 15]. Subgroup analysis found no significant difference in adverse reaction rates between the experimental and control groups for both ARS [RR = 0.26, 95% CI (0.03, 2.57), *P* = .25] and CRS [RR = 0.66, 95% CI (0.42, 1.05), *P* = .08]. Specific adverse events are detailed in Table 3.

**Table 3:** Occurrence of adverse reactions.

Literature sources	Adverse effect	
	T	C
Chen Juntao (2023)^[27]^	2 cases of rash, 2 cases of diarrhea, 2 cases of fever	6 cases of rash, 6 cases of diarrhea, 4 cases of fever
Liu Ying (2022)^[31]^	2 cases of epistaxis, 1 case of nasal irritation, 1 case of nausea and vomiting, 1 case of loss of appetite	3 cases of nosebleed, 2 cases of nasal irritation, 1 case of rash, 2 cases of loss of appetite
Sun Yi (2022)^[29]^	1 case of dizziness, 1 case of epistaxis	1 case of dizziness, 1 case of epistaxis, 1 case of nasal dryness
Wu Yumei (2021)^[35]^	2 cases of gastrointestinal reaction, 2 cases of oral odor	1 case of gastrointestinal reaction, 1 case of oral odor
Zheng Yongfeng (2022)^[15]^	1 case of nausea, 1 case of diarrhea	5 cases of nausea, 4 cases of vomiting, 5 cases of diarrhea, 4 cases of abdominal pain, 3 cases of loss of appetite
Chen Xiaoxia (2022)^[13]^	4 cases of nausea and vomiting, 4 cases of dizziness and headache, 3 cases of palpitation and irritability, 4 cases of drug eruption	2 cases of nausea and vomiting, 3 cases of dizziness and headache, 3 cases of palpitation and irritability, 3 cases of drug eruption
Yu Fengying (2020)^[18]^	None	3 cases of rash, 2 cases of nausea and vomiting, 1 case of diarrhea
Hou Linlin (2018)^[47]^	1 case of nausea, 1 case of vomiting, 1 case of dizziness, 1 case of mucous membrane thickening	1 case of nausea, 1 case of vomiting, 2 cases of dizziness, 1 case of mucous membrane thickening
Du Jingwei (2016)^[51]^	1 case of diarrhea, 1 case of rash, 1 case of nausea	1 case of nausea, 1 case of elevated transaminases
Jiang Liyuan (2015)^[56]^	1 case of diarrhea, 1 case of nausea and vomiting	1 case of nausea and vomiting, 1 case of abdominal pain, 1 case of dizziness

### 3.5. Sensitivity analysis

The sensitivity analysis was meticulously redesigned for clarity and precision. This involved the sequential omission of individual studies identified for their high variability to assess their individual impact on the aggregated outcomes. The exclusion of each study was followed by a recalculation of the pooled effect sizes to ensure the robustness of our findings. For the Lund-Kennedy score, the exclusion of the study by Hao Yanan from the ARS group^[14]^ led to the following results: [MD = ‐1.56, 95% CI (‐1.70, ‐1.43), *P* < .00001, *I*² = 6%]. This indicates that the overall effect size remains significant with low heterogeneity, suggesting that our findings are robust. In the SNOT-20 score, after the study by Gu Wei^[28]^ from the CRS group was excluded, the results were as follows: [MD = ‐3.67, 95% CI (‐4.25, ‐3.10), *P* < .00001, *I*² = 0%]. The absence of significant heterogeneity post-exclusion confirms the reliability of the pooled results. Regarding the VAS scores for symptoms of runny nose and olfactory impairment, the exclusion of Xu Wei study^[40]^ resulted in these outcomes: [MD = ‐0.70, 95% CI (‐0.91, ‐0.49), *P* < .00001, *I*²=49%] for runny nose and [MD = ‐1.31, 95% CI (‐1.52, ‐1.10), *P* < .00001, *I*² = 3%] for olfactory impairment. The moderate to low heterogeneity indices post-exclusion imply that the initial conclusions are not unduly influenced by any single study. In IL-6, after Sun Yi study^[29]^ from the CRS group was removed, the adjusted result was: [SMD = ‐1.24, 95% CI (‐1.56, ‐0.93), *P* < .00001, *I*² = 47%]. The elevated heterogeneity index suggests that the variability within the study may be attributed to differences in interventions as reported in the original literature. Lastly, for the occurrence of adverse reactions, the exclusion of Chen Xiaoxia study from the ARS group^[13]^ yielded: [RR = 0.09, 95% CI (0.03, 0.31), *P* = .0001, *I*²=0%]. The low heterogeneity and significant result post-exclusion indicate that the study’s findings are consistent across the remaining studies, highlighting the generalizability of the adverse reaction outcomes.

### 3.6. Publication bias detection

**Figure 16.**
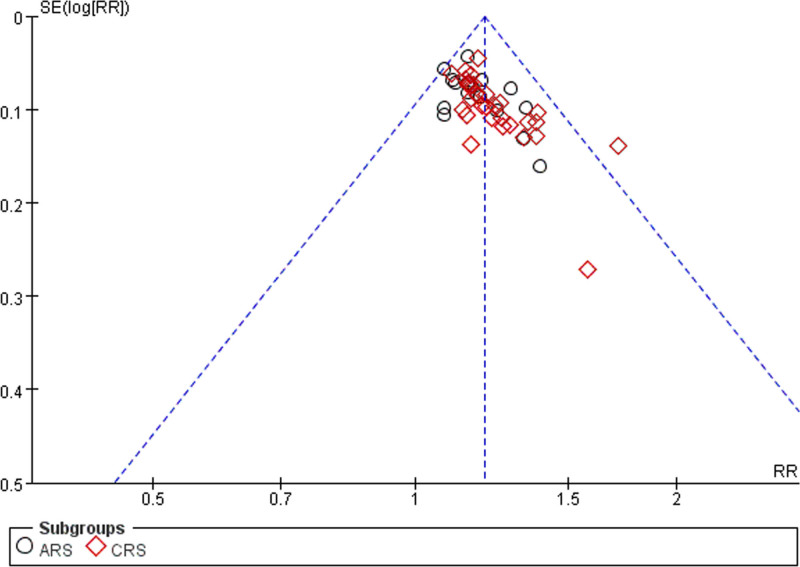
Funnel plot of total effective rate.

**Figure 17.**
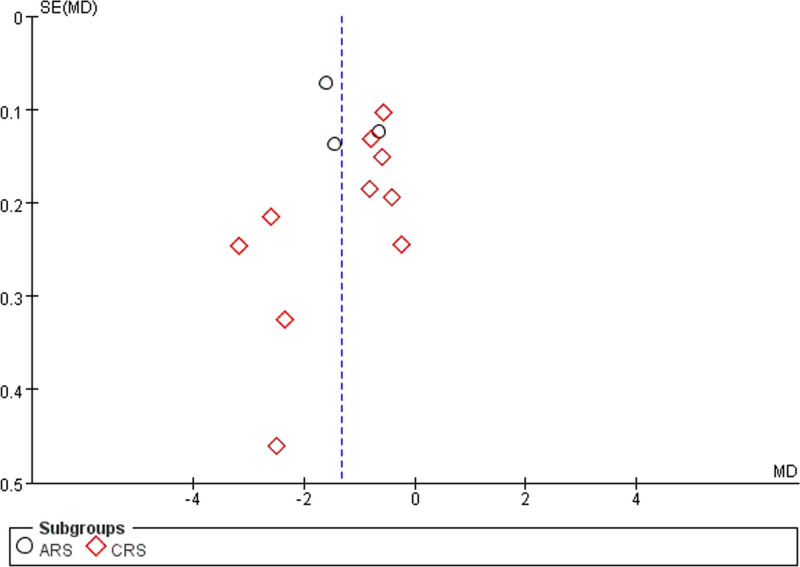
Funnel plot of Lund–Kennedy score.

**Figure 18.**
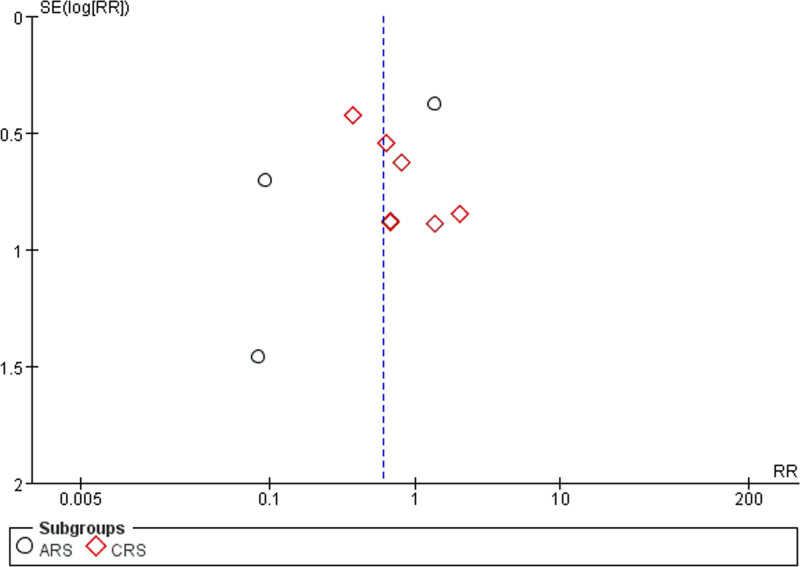
Funnel plot of adverse reactions.

Forty-eight articles^[10–57]^ reported information on the total response rate. Thirteen articles^[11,12,14,17,30,31,33,36,37,41–44,51]^ reported on the Lund–Kennedy score. Fourteen articles^[13,15,18,27,29,31,35,42,44,47,50,51,53,56]^ discussed the occurrence of adverse effects. Funnel plots were used to assess publication bias. The results indicated that the distribution of each scatter point exhibited asymmetry, which could be indicative of a bias in published works. The existence of this bias can be linked to the propensity of investigators to report favorable outcomes, the suboptimal quality associated with certain included investigations, and the limited participant numbers in some of the research conducted, as depicted in Figures 16–18.

### 3.7. Assessment of the quality of the evidence

**Figure 19.**
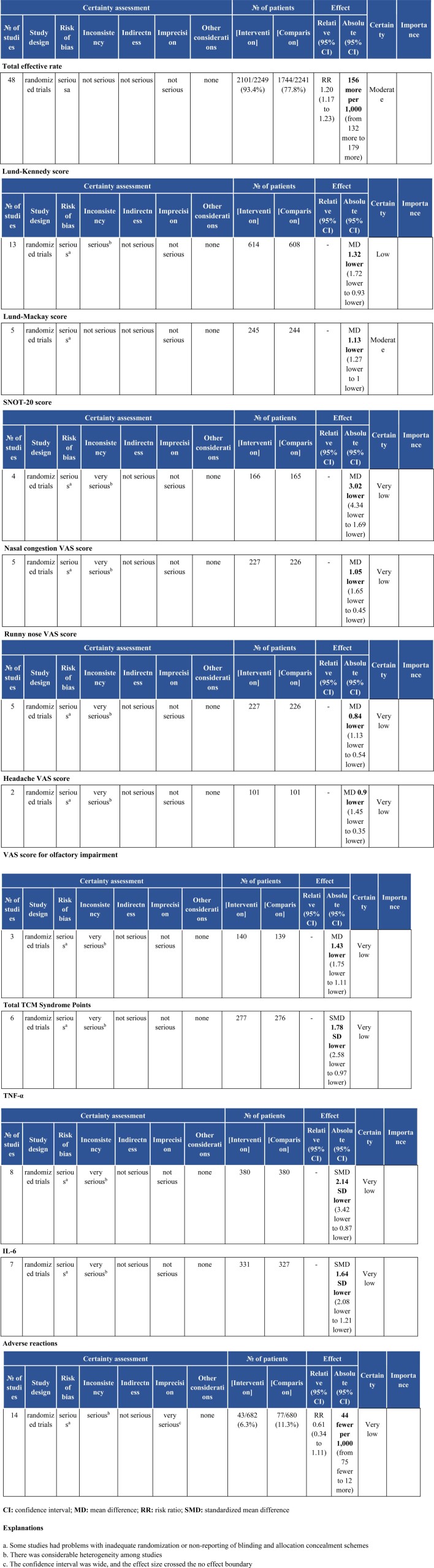
GRADEPro GDT quality assessment of outcomes.

The results of GRADEPro GDT were utilized to evaluate all outcomes, and the results indicated that the total effective rate and Lund–Mackay score were moderate-quality evidence, the Lund–Kennedy score was low-quality evidence, and the rest of the SNOT-20 score, nasal congestion VAS score, runny nose VAS score, headache VAS score, olfactory impairment VAS score, TCM syndrome total score, TNF-α, IL-6, and the incidence of adverse reactions were very low-quality evidence, see Figure 19A–G.

## 4. Discussion

RS is known as “nasal abyss” and “brain leakage” in ancient Chinese medicine literature. The earliest record of the pathogenesis of this illness^[58]^ is in the “Su Wen × Qi Convulsion Treatise”: “If the gallbladder is hot in the brain, then the nose will be abyss, and the nasal abyss will not stop under the turbidity.” Following the “Neijing,” doctors in the past dynasties have also discussed more about this disease, and the theory of Chinese medicine about the nose abyss is also constantly updated. The “14th Five-Year Plan” textbook for higher education in the national traditional Chinese medicine industry, “Otorhinolaryngology of Traditional Chinese Medicine,” posits that the manifestation of the nasal abyss primarily stems from the incursion of external evils, causing lesions of the lungs, spleen, stomach, and gallbladder. And the deficiency syndrome is mostly due to the deficiency of qi in the lungs and spleen, and the evil qi is trapped for a long time, which leads to lingering diseases, and it is divided into syndrome types such as pulmonary meridian wind heat, bile depression and heat, spleen and stomach dampness and heat, lung qi deficiency and cold, and spleen deficiency and dampness.^[59]^ According to the pathogenesis described above, the clinical treatment should focus on eliminating wind and reducing heat, removing excess bile heat, clearing heat and moisture, warming, and nourishing the lungs, strengthening the spleen, and reducing dampness, and invigorating qi and promoting circulation. The Chinese medications examined in this study encompass TCM prescriptions, self-formulated formulas, and proprietary Chinese medicines. Their specific effectiveness also adheres to the principles. Huang Yingrui, Cui Dong^[6,60]^ and their colleagues executed an extensive examination of the current scholarly works pertaining to the management of RS through the application of TCM practices. They focused on classic prescriptions, self-simulated prescriptions, and proprietary Chinese medications. Their findings indicate that TCM has a considerable therapeutic benefit in treating RS. However, both are reviews in nature, lacking a systematic approach to comprehensive analysis and quantitative evaluation of the results of multiple independent studies, and there is only one meta-analysis of TCM treatment in patients with ARS,^[7]^ and there is a lack of discussion on patients with CRS. Therefore, to more precisely assess the effectiveness and safety of TCM in addressing RS, it is essential to undertake additional thorough and meticulous systematic reviews and meta-analyses. These studies will offer a stronger foundation of evidence to guide clinical procedures.

### 4.1. Effectiveness

This study included a total of 48 RCTs that concentrated on the application of TCM for the management of RS. The effectiveness of TCM for treating RS was assessed using various outcome indicators, including the total effective rate, Lund-Kennedy score, Lund-Mackay score, SNOT-20 score, VAS score for nasal congestion, runny nose, headache, and olfactory impairment, total TCM symptom score, TNF-α, IL-6, and the incidence of adverse reactions. The synthesized findings from the meta-analysis indicated that the therapeutic effectiveness of integrating TCM with WMCT for RS surpassed that of WMCT alone. According to the subgroup analysis of the patients’ disease types, the experimental group showed good therapeutic effects in improving the total effective rate of patients with CRS, reducing the Lund-Kennedy score, Lund-Mackay score, SNOT-20 score, nasal congestion, runny nose, headache, olfactory impairment and other VAS scores, total TCM symptom score, TNF-α, IL-6 and other aspects. In the treatment of patients in the ARS group, the experimental group could improve the total effective rate and reduce the Lund–Kennedy score, TCM syndrome total score and IL-6, but in terms of reducing TNF-α, the experimental group’s performance did not diverge from that of the control group, and there was no evidence that the experimental group demonstrated improvements in the aforementioned metrics, including the Lund–Mackay score, SNOT-20 score, nasal congestion, runny nose, headache, and olfactory disturbance. Further clinical experimentation is essential to procure additional empirical support for employing TCM in the therapeutic management of RS patients.

### 4.2. Safety

Of the 48 included articles, 10^[13,15,18,27,29,31,35,47,51,56]^ reported specific adverse events, manifested as rash, nausea and vomiting, abdominal pain and diarrhea, nosebleeds, nasal irritation, dizziness, and headache. The synthesized data from the meta-analysis revealed a *P*-value of .10, suggesting that the observed differences did not reach a level of statistical relevance. This implies that the frequency of adverse reactions was similar between the TCM-enhanced western medical treatment and the standard western medical intervention, a finding that could be associated with the incorporation of western pharmaceuticals. Antibiotics are effective in controlling infections in the treatment of RS, but their irrational use may increase the incidence of adverse effects and increase the risk of resistance.^[61]^ Nasal hormones can reduce the inflammatory response of the nose, but their use will cause certain irritation to the nasal mucosa, which may lead to the rupture of tiny blood vessels in the nasal cavity, causing nasal irritation, nosebleeds, and other discomforts. Antihistamines can control allergic reactions in patients with CRS with allergic rhinitis, but the inherent pharmacological properties of these medications tend to precipitate unwanted side effects, including lightheadedness, cephalgia, and gastrointestinal unease.^[8]^ Cui Dong et al^[6]^ provided a synthesis of a decade’s worth of clinical studies on the application of classical prescriptions and proprietary Chinese medicines for the management of RS. The analysis revealed that, in comparison to western medicines, TCM exhibited a favorable safety profile in its treatment approach. However, in the specific practice of the application of TCM, we need to carry out syndrome differentiation and treatment according to the patient’s medical history and signs, observe the patient’s condition after medication, and if there are adverse reactions, they should be recorded and treated in time to ensure the safety of treatment.

### 4.3. Limitations

(1) Because of the search conditions’ limitations, the literature collection was done manually. Only randomized controlled trials with positive results were included, while trials with insignificant results may have been excluded, potentially leading to publication bias. (2) Out of the 48 literatures included, 3 were categorized based on the order in which they were visited, 1 was categorized using the odd and even grouping method, 18 studies did not specify the randomization method, only one article used the random envelope method for allocation concealment, only 2 cases were blinded to investigators and participants, and 2 outcomes had incomplete datasets and were exempt from the intention-to-treat analysis. The methodological rigor of the enrolled studies was suboptimal, potentially leading to the introduction of bias in the findings. (3) The collected literature featured variations in participant age and intervention strategies, with clear heterogeneity across the individual studies. Following subgroup analyses, the restricted volume of studies within certain subgroups may compromise the statistical potency and dependability of the resultant outcomes. (4) The literature search deadline is February 2024, and as clinical trials on the treatment of RS with TCM are still ongoing, there may be some studies published after the deadline for this analysis, which may have an impact on our results. (5) The included literature was all RCTs conducted in China, the language was Chinese, and there was a lack of large-sample, multicenter RCTs, which introduced language bias and regional bias, which limited the global applicability of this study.

### 4.4. Clinical guidance significance

The findings from this meta-analytical study indicated that the integration of TCM with standard western medicine for the management of RS yielded superior outcomes compared to western medicine alone. Additionally, the frequency of adverse events observed with this combined approach was comparable to that of the conventional western treatment, thereby highlighting the therapeutic efficacy and safety profile of TCM in this context. In the treatment of sinusitis, TCM takes comprehensive approach and treats the patient according to their symptoms and signs, removing the excess and making up for the deficiency, and finally achieving the effect of treating the disease.^[62]^ At present, in the guidelines for the treatment of sinusitis in China, only TCM treatment is mentioned in the guidelines for chronic sinusitis, but the specific syndrome types have not been listed and specific TCM or TCM protocols have not been recommended.^[8]^ In the future, numerous clinical investigations are essential for refining the criteria for various syndrome categories among RS patients and for instituting tailored consensus formulas to unify the therapeutic protocols for RS patients utilizing TCM.

## 5. Conclusion

In conclusion, the amalgamation of TCM with conventional western practices for RS treatment surpasses the efficacy of western medicine in isolation. This combination treatment improves the overall success rate of treatment, reduces scores related to nasal symptoms, such as congestion and runny nose, alleviates symptoms like headache and olfactory disorders, and decreases inflammatory indicators like TNF-α and IL-6. Additionally, the incidence of adverse reactions is comparable to that of WMCT. However, the interventions encompassed within this investigation exhibited considerable diversity, and there was significant disparity among the research conducted. The standard of methodology was subpar, with several studies omitting details regarding randomization procedures, allocation concealment, and blinding methods. Moreover, an intention-to-treat analysis was not conducted for incomplete outcome data, thereby compromising the dependability of the outcomes. For subsequent investigations, it is imperative to implement a stringent and uniform framework for clinical trials, with the execution of high-quality, multicenter RCTs to substantiate their effectiveness and safety. This approach will yield robust evidence-based medical support for the utilization of TCM in the treatment of RS patients.

## Author contributions

**Conceptualization:** Zhihao Huang, Xin Xuan, Shan Liu, Lifen Chen, Yanwen Cai.

**Data curation:** Zhihao Huang, Xin Xuan, Shan Liu, Lifen Chen, Ruoqing Qiu.

**Formal analysis:** Zhihao Huang.

**Funding acquisition:** Zhihao Huang, Shan Liu, Yanwen Cai.

**Investigation:** Zhihao Huang, Xin Xuan, Shan Liu, Yanwen Cai.

**Methodology:** Zhihao Huang, Xin Xuan, Shan Liu, Jinglan Lin, Yanwen Cai.

**Project administration:** Zhihao Huang, Xin Xuan, Shan Liu, Jinglan Lin, Zhijun Qian.

**Resources:** Zhihao Huang, Xin Xuan, Shan Liu, Jinglan Lin, Zhijun Qian.

**Software:** Zhihao Huang, Xin Xuan, Shan Liu, Jinglan Lin, Zhijun Qian, Lifen Chen, Ruoqing Qiu.

**Supervision:** Zhihao Huang, Xin Xuan, Shan Liu, Jinglan Lin, Zhijun Qian, Lifen Chen, Ruoqing Qiu.

**Validation:** Zhihao Huang, Xin Xuan, Shan Liu, Jinglan Lin, Zhijun Qian, Lifen Chen, Ruoqing Qiu, Yanwen Cai.

**Visualization:** Zhihao Huang, Xin Xuan, Shan Liu, Zhijun Qian, Lifen Chen, Ruoqing Qiu.

**Writing – original draft:** Zhihao Huang, Xin Xuan, Shan Liu.

**Writing – review & editing:** Zhihao Huang, Xin Xuan, Shan Liu.
